# 

**Published:** 1846-12

**Authors:** 


					T.
?
?? v.- 3tsS ? .-V .?? iSmiSS&tm
by John Tome* Esq., Surgeon Dentist,
Teeth from the Gullet
wmm
ana m
Hullihen,.
?;ssmsk^:&
?
-Introductory Lecture, before the Cla.. of the Baltimore
By A.
from a Free Incision of the Gums,.
CTAJJiE A. ?
W ffT^nr? v? -' w' - *n? ^3PPS
from
18SK
.' : .
?' ??II
193
?? ? ?? ?? ?    ;*'*?
193
......    .v....-V.....
. Paiaof Surgical Operations,..,.
Bfei "is&S&aS-&%
?V * J?R-
A Beautiful Cs
Irregularity of the Teeth,         197
Spiral Springs for Artificial Teeth,       197

				

